# Supplementary material for: Liquid-state quantitative SERS analyzer on self-ordered metal liquid-like plasmonic arrays
Source: Nat Commun. 2018 Sep 7;9:3642. doi: 10.1038/s41467-018-05920-z (PMC6128918; doi:10.1038/s41467-018-05920-z)
Supplement: Supplementary file 1 — Supplementary Information [file 41467_2018_5920_MOESM1_ESM.pdf]

Liquid-state quantitative SERS analyzer on self-ordered metal liquid-like plasmonic arrays

(Tian et al.)

Supplementary Information

Correspondence to liuhonglin@mail.ustc.edu.cn (H.L.); tan@chem.ufl.edu (W.T.)

Supplementary Methods. Assuming a cylindrical model of GNR with a length-to-diameter ratio of 3.0 based on TEM images, the formula *F (q)* is the form factor of cylindrical particles as

$$\mathrm{F}(\mathrm{q},\beta,r) = 2\mathrm{V}\rho_0 \frac{\sin(qHcos\beta)}{qHcos\beta} \times \frac{J_1(qRsin\beta)}{qRsin\beta}$$

where H is the height of the cylinder, R is the radius, β is the orientation angle, and our sample is random where q is the scattering wave vector, and $J_1(x)$ is a first order Bessel function. *ETA* is the center-to-center distance between particles, and *M* is the average scattering size of particles. The calculated *ETA* and *M* values are plotted as functions of volume of GNRs by the use of Irena software.

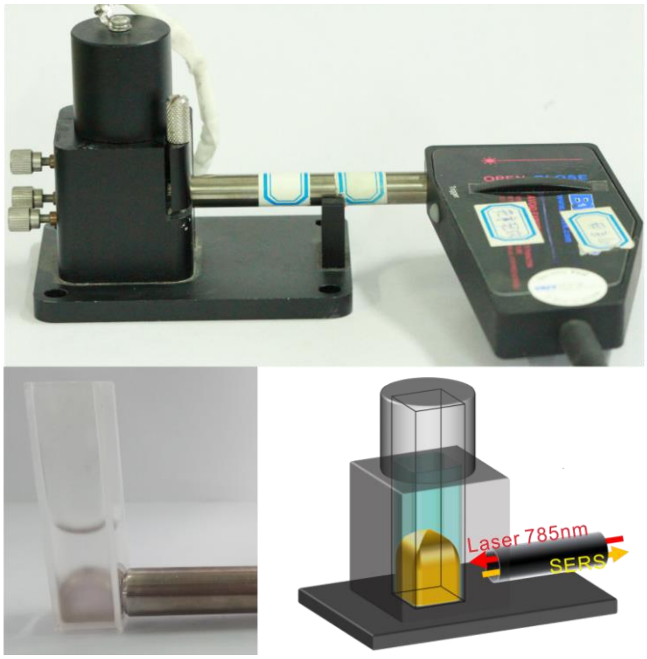

Supplementary Fig. 1 Illustrations of reversible *O/W* encasing for self-assembly of metal liquid-like GNR array, as a multiphase liquid-state SERS analyzer (Left), and the experimental setups of GNR array excited by fiber-optics probe on hand-held Raman device (Right).

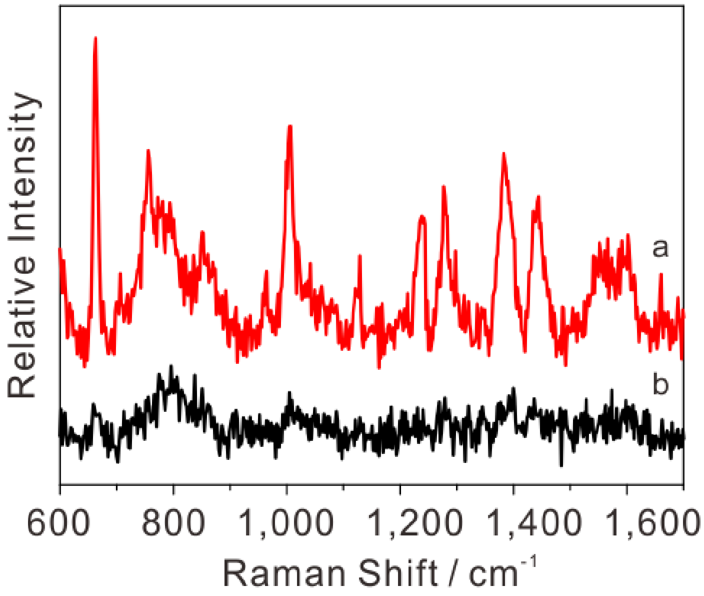

Supplementary Fig. 2 SERS spectrum of the top interfacial GNR film (a) and Ci-GNR sols (b).

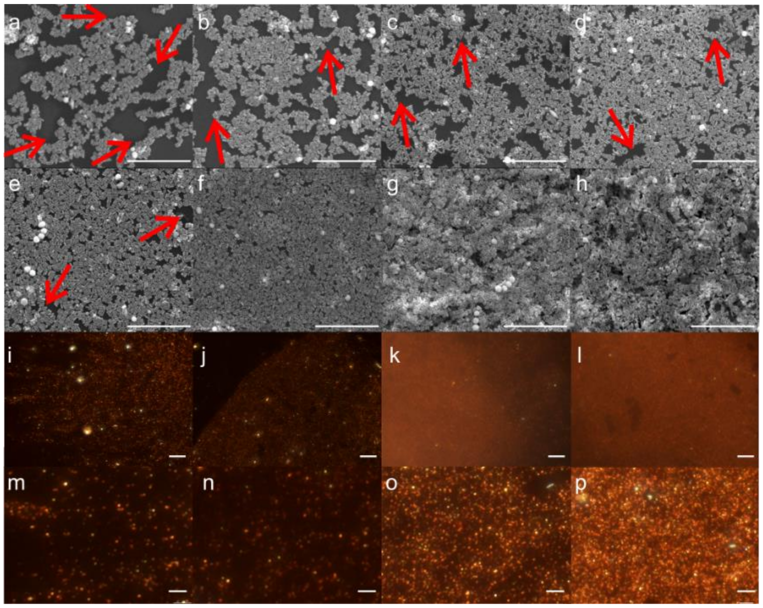

Supplementary Fig. 3 (a-h) SEM images of metal liquid-like arrays fabricated by 1 mL GNR sols with different OD values: 1.0, 2.2, 3.4, 4.6, 5.8, 7.0, 8.2, 9.4, and all of the scale bars are 1 μm. (i-l) DFM images of diluted interfacial arrays fabricated by 1 mL GNR sols with different OD values: 0.3, 0.6, 0.9, 1.2, and all of the scale bars are 20 μm. (m-p) Enlarged detail of the corresponding DFM images in i-l, and all of the scale bars are 5 μm.

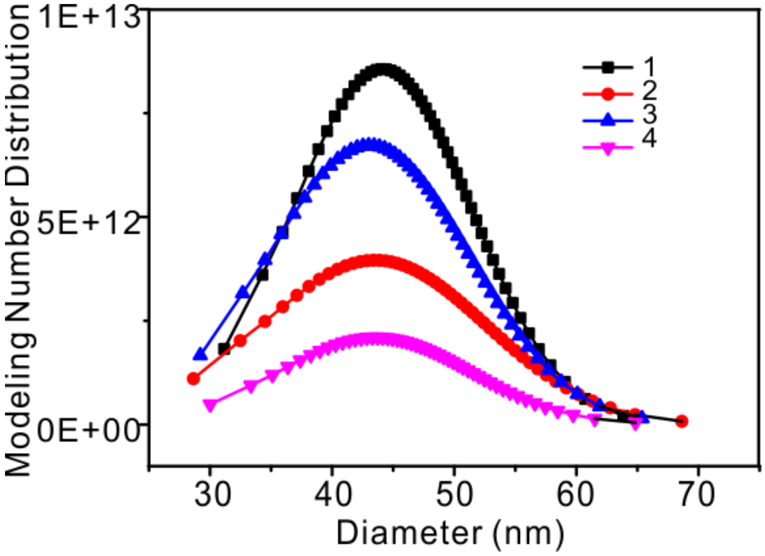

Supplementary Fig. 4 Calculated particle size distribution from SR-SAXS data of four different samples: 1, as-synthesized GNR sols of 3 OD; 2-4, interfacial arrays fabricated by GNR sols with varied OD values: 3.0, 6.0, and 9.0, respectively.

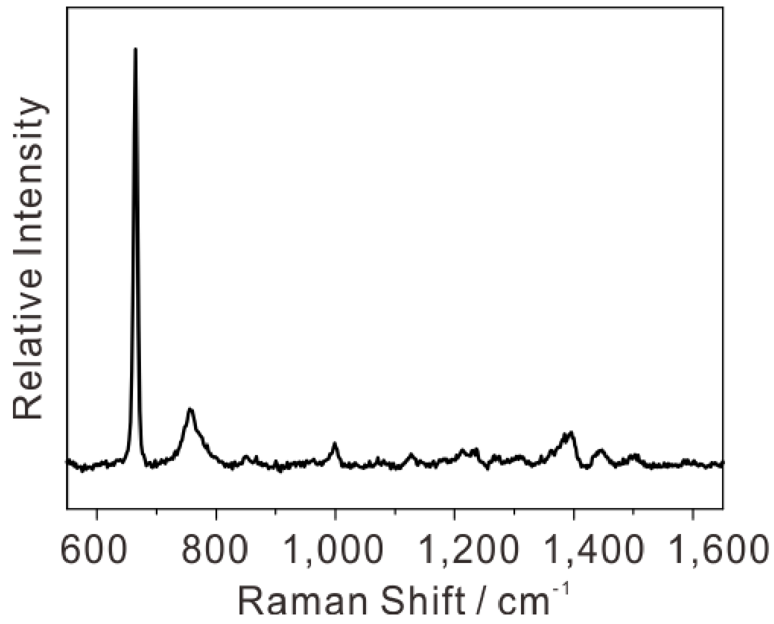

Supplementary Fig. 5 SERS spectrum of chloroform collected on liquid interfacial GNR arrays.

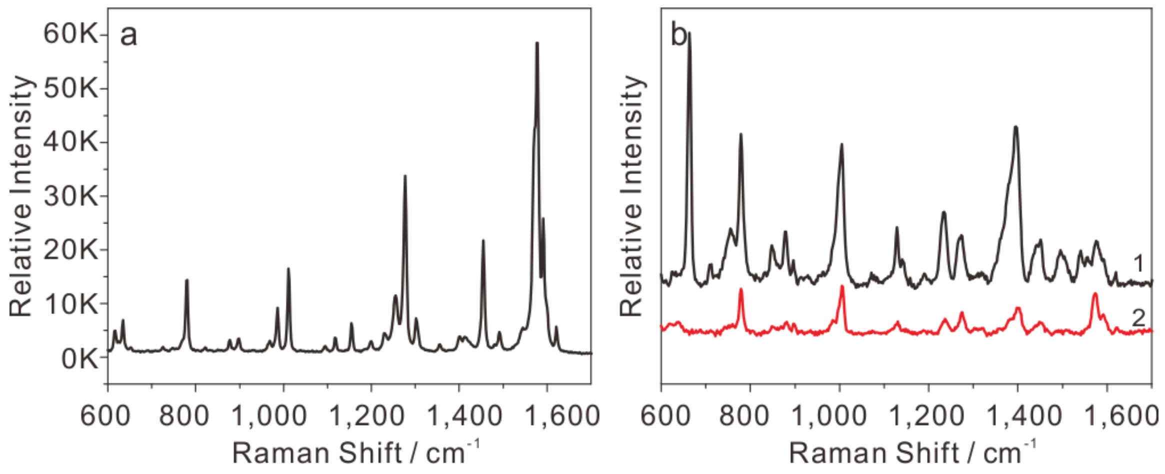

Supplementary Fig. 6 (a) Raman spectrum of TBZ powder. (b) SERS spectra of TBZ on (1) the liquid interfacial GNR arrays and (2) the solid GNRs on Si wafer, respectively.

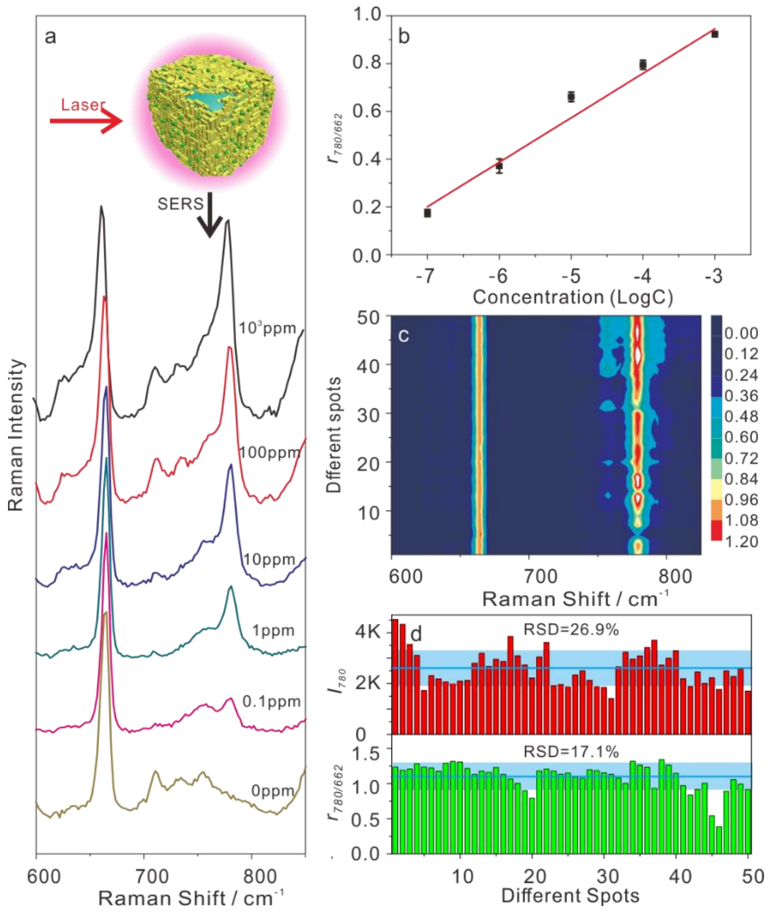

Supplementary Fig. 7 (a) Metal liquid-like GNR arrays on *W*-in-*O* interface for SERS analysis of TBZ with concentrations of 0, 0.1, 1, 10, 100, and 1,000 ppm, respectively, dissolved in the *O* phase. (b) A linear relationship between the $r_{780/662}$ values and the logarithmic TBZ concentrations. The error bars represent the statistical RSD and were calculated from five different runs. (c) Fifty runs in triplicate SERS experiments generating a 2D spectral mapping of 1000 ppm TBZ. (d) Statistical histograms of $r_{780/662}$ and I_{780}, respectively.

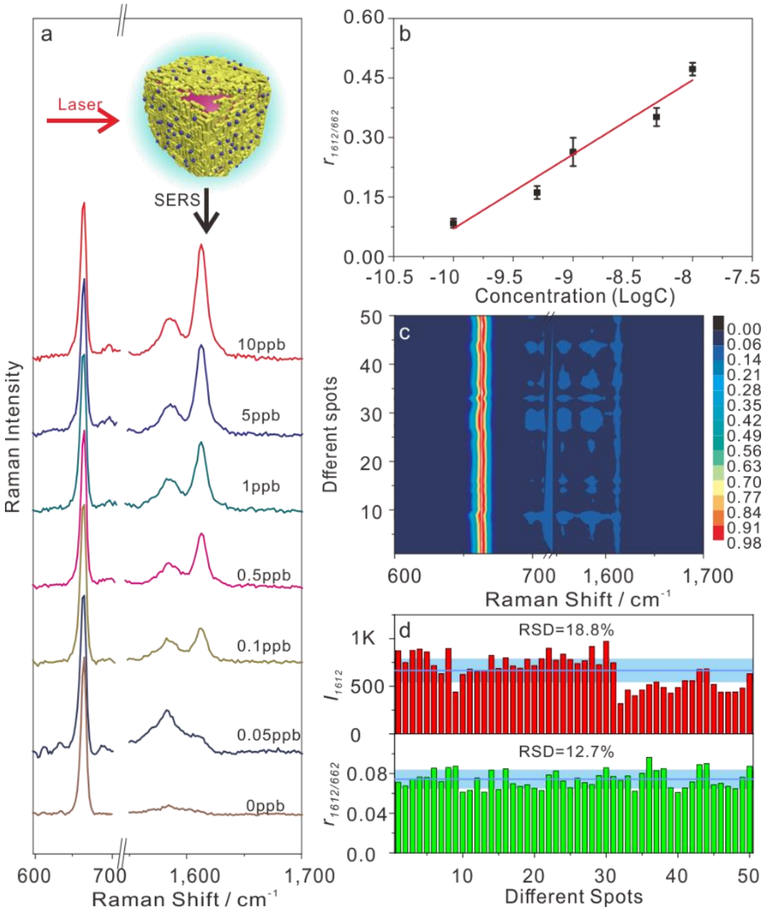

Supplementary Fig. 8 (a) *O*-in-*W* platform for SERS analysis of MG dissolved in the *W* phase with concentrations of 0, 0.05, 0.1, 0.5, 1, 5, and 10 ppb, respectively. (b) A linear relationship between the $r_{1612/662}$ values and the logarithmic MG concentrations. The error bars represent the statistical RSD and were calculated from five different runs. (c) Fifty runs in triplicate SERS experiments generating a 2D spectral mapping of 0.5 ppb MG. (d) Statistical histograms of $r_{1612/662}$ and I_{1612}, respectively.

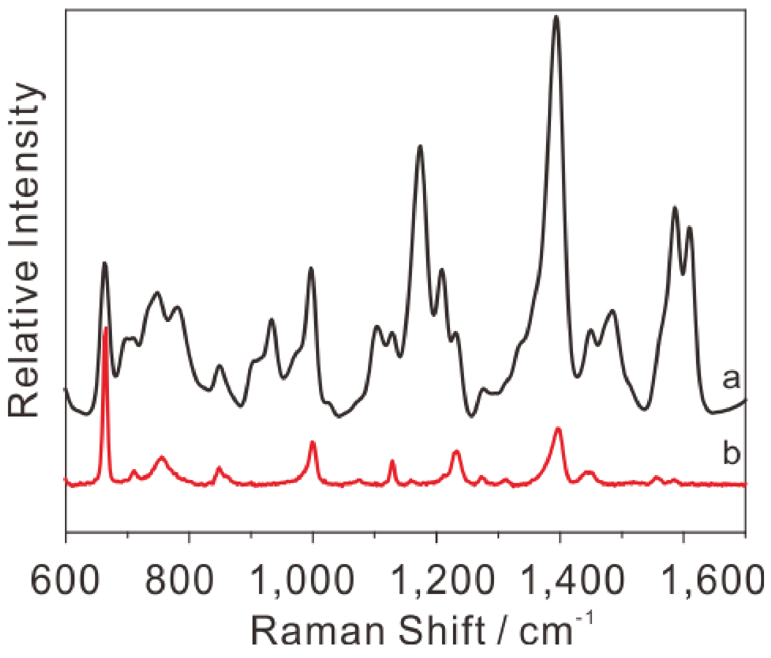

Supplementary Fig. 9 SERS spectra of MG (a) and blank sample (b).

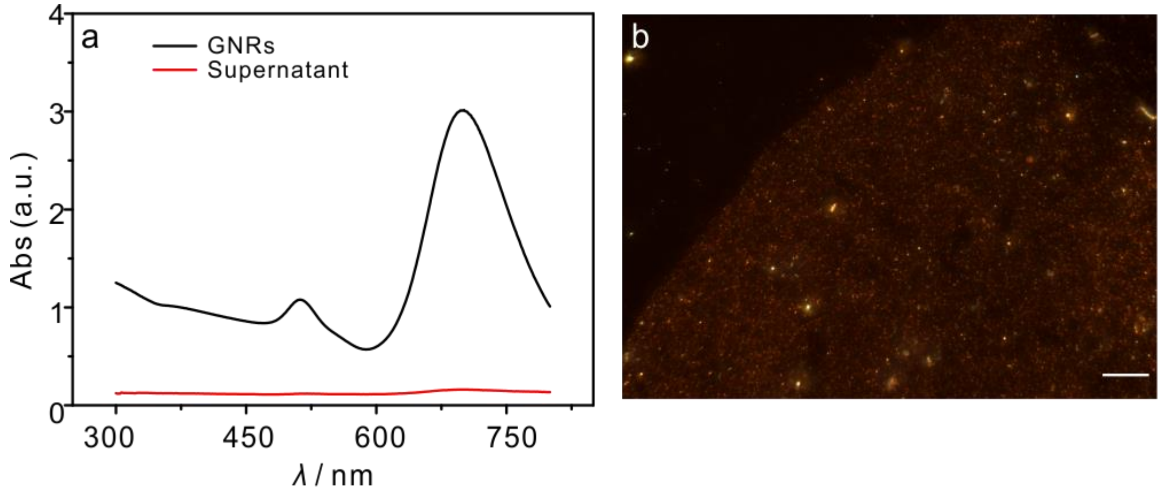

Supplementary Fig. 10 (a) UV-Vis absorbance spectra of the aqueous phase before and after interfacial assembly with 1 mL of 7.5 OD GNR sols. (b) DFM observations on the edge of metal liquid-like droplet fabricated with 0.6 OD GNRs, and the scale bar is 20 μm.

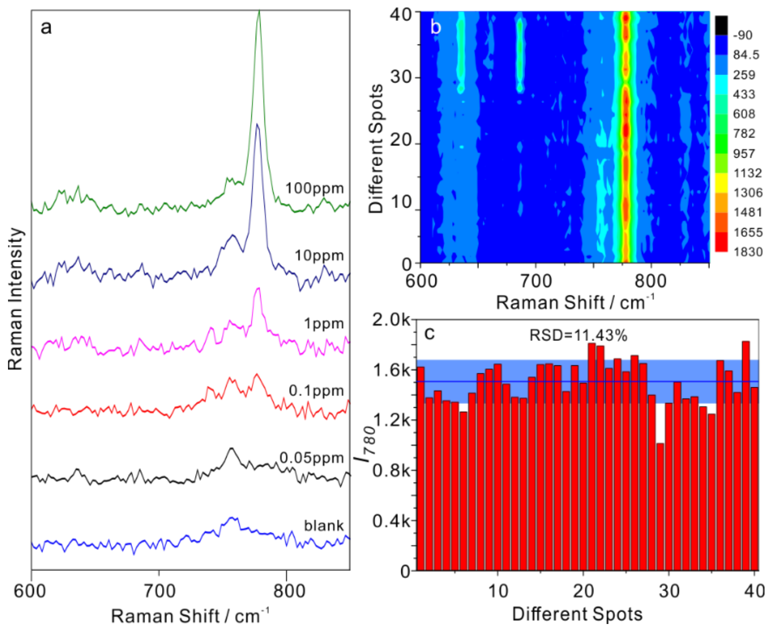

Supplementary Fig. 11 SERS performance comparison between the typical solid substrates of GNRs and our metal liquid-like interfacial GNR arrays. (a) The SERS spectra of TBZ with different concentrations on the solid aggregates of GNRs on Si wafer, (b) Forty random runs in triplicate SERS experiments generating a 2D spectral mapping of 100 ppm TBZ. (c) Statistical histograms of I_{780} collected on Si wafer solid platform.

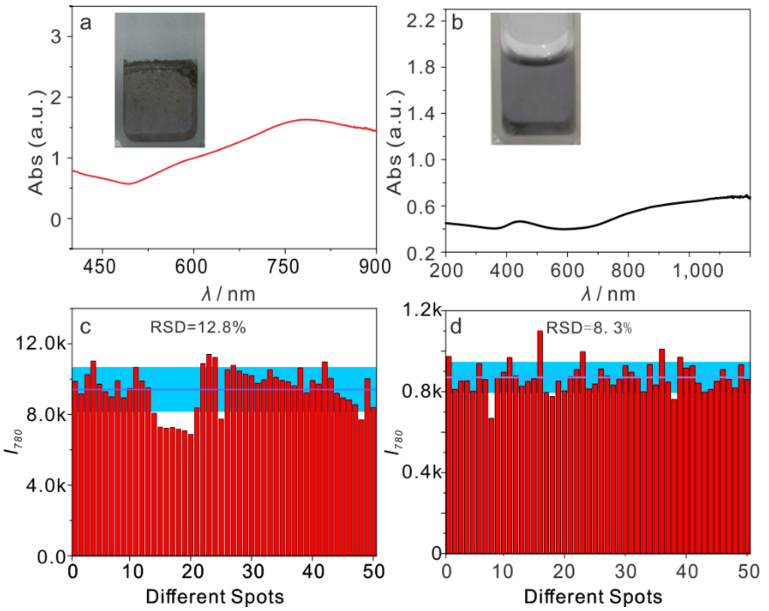

Supplementary Fig. 12 SERS performance comparison between the salt-induced aggregates of GNR sols and our metal liquid-like interfacial GNR arrays under similar conditions. Comparison between (a and c) *O*-in-*W* metal liquid-like GNR arrays and (b and d) salt-induced aggregates of GNR sols with respect to UV-Vis absorbance, optical topography, and SERS intensity histograms at 780 cm^{-1} (I_{780}) without IS calculations. Note: Two platforms used the same 1 mL of 6.0 OD GNR sols. The aggregate of GNR sols was produced by adding 0.5 M of sodium chloride, and this resulted in a homogeneous as-prepared aggregating system, showing a deep-blue color, that could stabilize within a half hour. The analyte TBZ had a final concentration of 10 ppm in *O*-in-*W* interfacial GNR arrays, but 100 ppm in salt-induced aggregates of GNR sols. Moreover, the excitation laser was 60 mW for interfacial GNR arrays, but 90 mW for aggregated GNR sols.

Supplementary Table 1 Surface wettability effects on TBZ Raman bands.[1]

Theoretical / cm^{-1}	Experimental / cm^{-1}		Assignments
	O-in-*W*	*W*-in-*O*	
785	780	780	C–H bending
1,019	1,006	1,006	C-C-C bending
1,285	1,272	1,272	Ring stretching
1,549	1,540	1,540	C=N stretching
1,583	1,571	1,571	C-C stretching

Supplementary references

1. He, L., Chen, T. & Labuza, T. P. Recovery and quantitative detection of thiabendazole on apples using a surface swab capture method followed by surface-enhanced Raman spectroscopy. *Food Chem.* **148**, 42-46 (2014).
