# Supplementary material for: Liquid-state quantitative SERS analyzer on self-ordered metal liquid-like plasmonic arrays
Source: Nat Commun. 2018 Sep 7;9:3642. doi: 10.1038/s41467-018-05920-z (PMC6128918; doi:10.1038/s41467-018-05920-z)
Supplement: Supplementary file 3 — Description of Additional Supplementary Files [file 41467_2018_5920_MOESM3_ESM.pdf]

Descriptions of Additional Supplementary Files

File Name: Supplementary Movie 1
Description: Three cycles of reversible O/W transition of metal liquid-like GNR arrays in cuvettes with hydroxylated and fluorosilylated treatments.
